# Navigating meaningful engagement: lessons from partnering with youth and families in brain-based disability research

**DOI:** 10.1186/s40900-024-00543-9

**Published:** 2024-02-05

**Authors:** Linda Nguyen, Kinga Pozniak, Sonya Strohm, Jessica Havens, Claire Dawe-McCord, Donna Thomson, Connie Putterman, Dana Arafeh, Barb Galuppi, Alicia Via-Dufresne Ley, Shelley Doucet, Khush Amaria, Adrienne H. Kovacs, Ariane Marelli, Ronen Rozenblum, Jan Willem Gorter, CHILD-BRIGHT READYorNot™ Brain-Based Disabilities Trial Study Group

**Affiliations:** 1 School of Physical and Occupational Therapy, Faculty of Medicine and Health Sciences, McGill University, Montreal, QC Canada; 2 CanChild Centre for Childhood Disability Research, McMaster University, Hamilton, ON Canada; 3 Patient and Family Advisory Council, CanChild Centre for Childhood Disability Research, McMaster University, Hamilton, Canada; 4 The Research Institute of the McGill University Health Centre (RI-MUHC), Montreal, QC Canada; 5 Nursing and Health Sciences, University of New Brunswick, Saint John, NB Canada; 6 CBT Associates (A CloudMD Company), Toronto, ON Canada; 7 Equilibria Psychological Health, Toronto, ON Canada; 8 Department of Medicine, Faculty of Medicine, McGill University, Montreal, QC Canada; 9 Division of General Internal Medicine and Primary Care, Department of Medicine, Brigham and Women’s Hospital, Boston, MA USA; 10 Harvard Medical School, Boston, MA USA; 11 Department of Rehabilitation, Physical Therapy Science and Sports, UMC Utrecht Brain Center, University Medical Center Utrecht, Utrecht, The Netherlands; 12 Centre of Excellence for Rehabilitation Medicine, University Medical Center Utrecht and De Hoogstraat Rehabilitation, Utrecht, The Netherlands

**Keywords:** Patient engagement, Patient-oriented research, Patient-centered care, Authentic engagement, Adolescent and young adult, Families or caregivers, e-health, Healthcare transition, Brain-based disabilities

## Abstract

**Background:**

While patient and family engagement in research has become a widespread practice, meaningful and authentic engagement remains a challenge. In the READYorNot™ Brain-Based Disabilities Study, we developed the MyREADY Transition™ Brain-Based Disabilities App to promote education, empowerment, and navigation for the transition from pediatric to adult care among youth with brain-based disabilities, aged 15–17 years old. Our research team created a Patient and Family Advisory Council (PFAC) to engage adolescents, young adults, and parent caregivers as partners throughout our multi-year and multi-stage project.

**Main body:**

This commentary, initiated and co-authored by members of our PFAC, researchers, staff, and a trainee, describes how we corrected the course of our partnership in response to critical feedback from partners. We begin by highlighting an email testimonial from a young adult PFAC member, which constituted a “critical turning point,” that unveiled feelings of unclear expectations, lack of appreciation, and imbalanced relationships among PFAC members. As a team, we reflected on our partnership experiences and reviewed documentation of PFAC activities. This process allowed us to set three intentions to create a collective goal of authentic and meaningful engagement and to chart the course to get us there: (1) offering clarity and flexibility around participation; (2) valuing and acknowledging partners and their contributions; and (3) providing choice and leveraging individual interests and strengths. Our key recommendations include: (1) charting the course with a plan to guide our work; (2) learning the ropes by developing capacity for patient-oriented research; (3) all hands on deck by building a community of engagement; and (4) making course corrections and being prepared to weather the storms by remaining open to reflection, re-evaluation, and adjustment as necessary.

**Conclusions:**

We share key recommendations and lessons learned from our experiences alongside examples from the literature to offer guidance for multi-stage research projects partnering with adolescents, young adults, and family partners. We hope that by sharing challenges and lessons learned, we can help advance patient and family engagement in research.

## Abstract (plain language summary)

In the READYorNot™ Brain-Based Disabilities Study, our research team knew that for youth with disabilities, transitioning from children’s services to adult health services was difficult. So, we created an application to help disabled youth between 15 and 17 years old learn how to navigate the adult healthcare system. From the beginning of the study, researchers worked closely with youth and families in a Patient and Family Advisory Council (PFAC). This paper is initiated and co-authored by members of the PFAC and researchers. We wanted to think about and learn from a “critical turning point” when a young adult partner sent an email describing some real worries about working on this project. The young adult partner reported feeling “invisible”, “not heard” and felt that roles of members of the PFAC were not clear. This led to frustration and confusion—feelings shared by other members of the PFAC. This email led everyone on the project to think about working together differently. Researchers and PFAC members came together and agreed on new ways to partner: to offer clarity and flexibility around the roles of PFAC members, to value and appreciate partner contributions and to provide opportunities for all partners to contribute according to their interests and strengths. It was hard to realize that our team made mistakes, but we came together to learn and be an example for other research teams who face similar challenges.

## Background

### Embracing patient and family engagement

The engagement of patients and families in health research is becoming an expected practice [1]. Patients can be broadly defined as individuals with personal experience of a health condition, and their family [1]. The perspectives of patients and families can help ensure that research is meaningful, relevant, and applicable to improving patient outcomes [1–3]. Partners can be involved in all phases of research, from initial design (e.g., consulting on the protocol, setting research objectives, developing and piloting interview guides and questionnaires, informing consent processes [4, 5]), to recruiting and retaining participants [6], through to data analysis and knowledge translation. Moreover, when research concerns the development of new health technologies (e.g., digital applications or apps), partners can help inform the design, identify unmet needs, and tailor the technology to the users, thus optimizing usability and adherence [7].

### Principles for forming genuine partnerships with patients and families

Overarching ethical considerations in research relationships include reciprocity (i.e. ensuring that exchanges are based on mutual benefit and respect) and having a shared commitment to producing results that are relevant to improving health [8–10]. Patients must be treated as essential partners and appropriately supported, recognized, and compensated for their contributions. Studies have highlighted the importance of involving partners at early stages of research; properly initiating and orienting them to the project, and providing training and education to researchers, patients, and families on research design, statistics, patient engagement, and effective communication [11, 12]. There should also be ongoing reassessment and feedback throughout the research process [12]. When partners are meaningfully engaged and aware of how their perspectives and feedback are incorporated into research, they report feeling valued and validated [4–6, 13, 14], confident as experts in their lived experiences [5, 15], and proud of their contributions [13].

### Barriers and pitfalls to patient and family engagement in research

Commonly reported barriers to engagement include additional time and staff resources, time constraints for both partners and researchers, and funding required to support partners in the engagement processes [2, 5]. When adolescents and young adults (AYA) are engaged in research, they are most often consulted through the development and evaluation stages of health interventions, and less often truly engaged as partners [16]. Developing relationships with AYA and providing training could increase engagement in research [17]. Increasing expectations for researchers to include partners in research, without proper preparedness for engagement, can lead to tokenism or a false sense of inclusion [2, 18, 19]. Furthermore, there is limited guidance on how to engage with both AYA and parents together in a single advisory council and over the course of a multi-year project, and how to maintain engagement over time [20].

### The READYorNot™ brain-based disabilities (BBD) project

The READYorNot™ Brain-Based Disabilities Study was a project in the CHILD-BRIGHT national research network funded by the Canadian Institutes of Health Research’s Strategy for Patient-Oriented Research to improve outcomes for children with brain-based developmental disabilities and their families [21]. Transition from pediatric to adult healthcare systems is a critical milestone for a growing population of youth with lifelong conditions [22, 23]. The goal of healthcare transition is to maximize lifelong functioning and potential through the provision of uninterrupted healthcare services as individuals move from adolescence to adulthood [22, 23]. In the CHILD-BRIGHT READYorNot™ BBD project, we aimed to support this goal by developing an App to promote education, empowerment, and navigation to help youth manage their healthcare. The App’s intended users were youth with brain-based disabilities, with conditions such as autism spectrum disorder (ASD), cerebral palsy (CP), epilepsy, fetal alcohol spectrum disorder (FASD) or spina bifida, aged 15–17 years old. The project entailed developing the MyREADY Transition™ BBD App (the “App”) to test in a randomized controlled trial (RCT) to see if it improves transition readiness among the youth, and integrated knowledge translation.

### Our research team and Patient and Family Advisory Council (PFAC) experiences

The research team was multidisciplinary and included researchers, project staff, healthcare providers, technology experts, as well as AYA and parent partners. In the first stage of the project, three distinct groups were established to oversee aspects of the project including (a) an “IT” (e-health information technology) group to oversee creation of the App; (b) a “Content” group to develop a psychology-informed educational curriculum; and (c) an “Engagement” group responsible for capturing partner input and user experiences. The team also had a Patient and Family Advisory Committee (PFAC), composed of AYA and parent partners. Throughout the project, the PFAC met regularly with representatives from each of the project’s three groups, working most closely with the engagement group.

The PFAC was established in 2017 with the support of one parent and one AYA, and within several months had grown to include five parents and two AYA partners. In 2023, a total of 14 partners (eight AYA and six parents) had actively contributed to the PFAC over the course of the project, spanning six years. PFAC partners joined the project in a variety of ways, including by invitation (e.g., those who had pre-established relationships with members of the research team or who had partnered on previous projects), self-referral (e.g., those who contacted the research team after hearing or reading about the project on the CHILD-BRIGHT Network website).

Initially, PFAC meetings were held biweekly during daytime hours and centered around consulting on the design and content for the App. The meetings were conducted using a teleconferencing phone line with materials typically shared in advance by email or during meetings using a web-based screen-sharing application (e.g., https://www.screenleap.com) [24].

### Catalyst to reflect on our partnership processes

A year into this project in February 2018, one of the research coordinators on our team received the following email from a young adult patient partner, in which the patient partner voiced the following experience with partnering on our study: *“This is a rather heavy email… I feel like I am doing invisible work… I do not feel like my time or efforts are being respected… Am I the right person for the project?”*. This email served as a catalyst for our team to begin reflecting together about our partnership processes and implementing strategies moving forward [25]. With permission from the young adult and the research coordinator (both of whom are co-authors of this article), we use excerpts from this email in this paper to demonstrate how we reflect on our partnership journey. Researchers partnered with adolescent and young adult (AYA) patients and parents in this childhood disability research project to co-create and test an e-health intervention. This commentary is co-authored by several members of the CHILD-BRIGHT READYorNot™ BBD project’s PFAC, researchers, project staff, and a trainee. We present excerpts from the email testimonial which illuminated key challenges and led to changes in our collective team approach to partnering together [25]. Drawing on relevant excerpts from the email, as well as from experiences shared by the authors and other members of the research team, we describe how the email led to more deliberate attempts toward achieving meaningful, productive, and mutually beneficial engagement. We present the three intentions we set for working together towards authentic and meaningful engagement and the specific strategies we employed over the remainder of the project. Setting an intention involves collectively creating a goal and a vision of where we wish to go and charting a course to help us get there.

The objective of this paper is to describe practical guidance for researchers to consider incorporating when engaging AYA and parents as members of a PFAC. We hope that by sharing our own challenges, we add to the evidence base of useful lessons learned from patient and family engagement in research.

### Methods: Our approach

In late 2020, the “Engagement” group—including the PFAC—identified a desire to prepare this manuscript, documenting our lessons learned in partnering together. In early 2021, we established a writing sub-group comprised of project staff and researchers as well as three parents and three AYA partners. Members of this group met quarterly over the next two years to reflect on the actions we have taken in response to the email, and have organized these actions around three key sets of activities which we describe as our “intentions”. We also developed a set of recommendations grounded in our collective experiences. We subsequently invited all members of the “Engagement” group and wider research team to reflect on their experiences partnering together over the course of the project, and to consider the following questions: (1) What do you feel have been the strengths of our partnership?; (2) What have been some of the effective strategies used to engage PFAC members over time?; (3) What stands out to you as something that has evolved over time to make our partnership better?; and (4) In what ways can we work to improve things? These questions were proposed by the parent PFAC member (JM) who served the role of liaison between the larger research team and the PFAC. The questions were circulated as a fillable form and the responses were collated and discussed by the author team in a paper planning meeting. We also included an open-ended question to ask about experiences from the team about our partnership. Several PFAC members contributed ideas about what the partnership meant to them or what they felt that they “got out of” the experience. In addition to collecting these reflections, the subgroup reviewed meeting minutes, notes, and correspondences from PFAC meetings, emails, and individual check-in meetings. As part of our reflection process, we reviewed notes from a “Stop, Start, Continue” [26] activity that elicited ideas about what we should stop doing (e.g., what was not working for the team, what was not having desired outcome), what we should start doing (e.g., what new ideas did the team have, what could we do to address new situations), and what we should continue doing (e.g., what is working well for the team, what processes are successful). 

Together, we pivoted from the critical turning point towards more authentic and meaningful engagement, by working together to plan and implement the research trial, and to collaboratively interpret and disseminate results. In the following sections we present our experiences in the context of this email, drawing on relevant excerpts to describe our challenges, and to share what strategies we have found to be most effective for achieving the three intentions we set for ourselves.

## RESULTS: Three intentions we set for authentic and meaningful engagement and strategies used

**Fig. 1:**
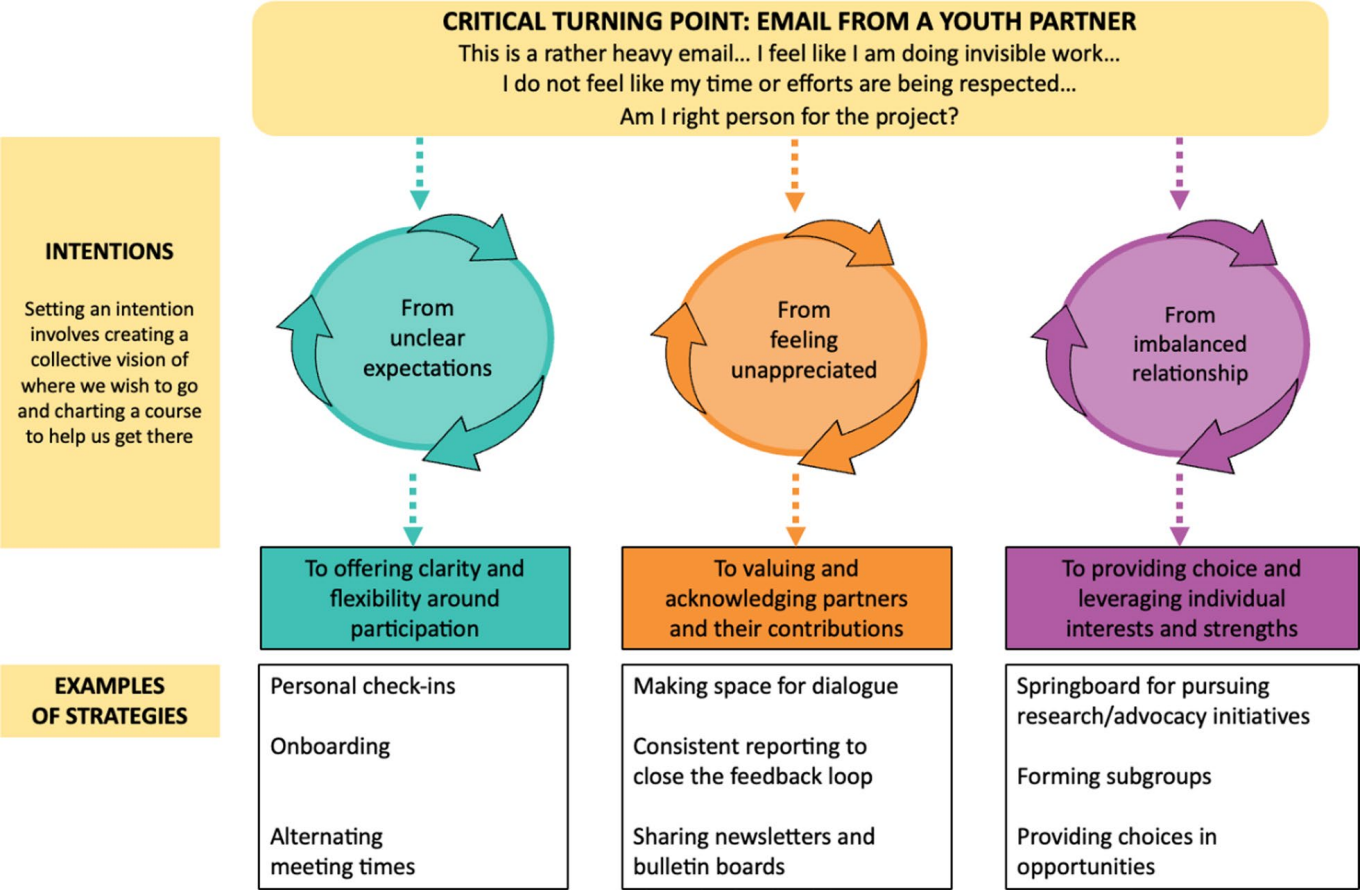
Three intentions we set for authentic and meaningful engagement and strategies we used

Many concerns raised in the AYA partner’s email resonated with other members of the PFAC. These issues also resonated with researchers and project staff who valued the importance of building a culture of engagement for AYA and parent partners but who were embarking on a new project with a new team and felt the pressure of the project timelines. As this project entailed both the development and a trial of an e-health intervention, it was marked by tight deadlines and a complex team structure. The accelerated pace at which different teams were working to meet technology development timelines often impeded the ability to carry out best practices of patient-oriented research and also took time away from the important foundational work of building relationships among the AYA, parent partners, and wider research team. As conveyed in the AYA partner’s email, this led to confusion around role expectations as well as to feelings of being unappreciated and not being equally and meaningfully involved. This inspired us to set three intentions for authentic and meaningful engagement, which are summarized along with associated strategies in Fig. 1 and are more fully described below.

### Intention #1: From unclear expectations toward offering clarity and flexibility around participation

“There was no orientation, manual, terms of reference, or documents that actually describe my role… It does not help that I am not able to participate in meetings because they directly interfere with my university classes. I need to know what I am supposed to be doing and the best way to do it remotely.”—Excerpt from email.

As individuals were welcomed onto the PFAC during the early stages of the project, they found themselves immediately thrust into tasks without having been appropriately introduced to the project or to their role. While an initial Terms of Reference had been developed and discussed at an early team meeting, this document was not reviewed with new members. This lack of onboarding was evidenced in the email testimonial, where the AYA partner expressed that they were not properly oriented, did not fully understand their role, and were unsure about how they could fully participate given that they were often unable to attend meetings due to conflicting school obligations. The inconvenience of meetings being scheduled during the day was a barrier for several individuals on the project.

In response to this feedback, it was important to our team to offer both clarity and flexibility around participation. We worked together to refine a Terms of Reference document to clarify role expectations and add important information such as CHILD-BRIGHT’s guidelines for patient-partner compensation [27]. This living document was shared and revisited with anyone who subsequently joined the PFAC, as part of an improved and more personalized onboarding process. Rotating the meeting times to accommodate different schedules and time zones, and shifting to video conferencing improved accessibility to attend meetings. Though the COVID-19 pandemic declared by the World Health Organization on March 11, 2020 [28] impacted our project in many ways, it is a testament to our partnership that the pandemic had minimal impact on how we were able to work together. We made it a priority for our project to invest in relationship- and community-building as we worked together toward a shared purpose. The opportunity to continue meeting virtually facilitated our sense of connection.

Researchers also acknowledged and supported partners to flexibly engage with the PFAC as their time permitted. For example, when partners stepped back at times due to health reasons or other commitments (e.g., school, family). Researchers implemented strategies to keep partners feeling connected, such as by recording meetings and using a shared drive where these recordings could be accessed along with other meeting materials and updates about ongoing and completed PFAC tasks and activities. Following the email, researchers introduced individual check-ins with PFAC members which took place bi-annually to review how they felt about their level of involvement and to explore how well their personal interests were being met. With the introduction of each new project task, we used a tool called the Involvement Matrix [24] to create clarity and to demonstrate flexibility and choice with respect to one’s preferred levels of involvement. The Involvement Matrix [29] is a tool to promote collaboration with AYA and parent partners in research, by aiding in the dialogue about the role that partners wish to play in the various activities of the project. It describes a continuum of roles from Listener (given information), Co-thinker (asked to give opinion), Advisor (gives (un)solicited advice), Partner (works as an equal partner), to Decision-Maker (takes initiative, makes final decisions). We also worked together to tailor ways of participating to PFAC preferences and needs. For example, when requesting feedback on documents or manuscripts, researchers offered one-on-one video calls for those who were less comfortable using track changes in a Microsoft Word (Version 16.71) document. When co-presenting with partners, researchers offered asynchronous opportunities, such as pre-recording contributions to a presentation if they were unable to attend a live event. In advance of meetings, researchers provided clear, concrete meeting objectives and highlighted priority items to help PFAC members prepare and decide if a meeting would be relevant to their role.

### Intention #2: From partners feeling unappreciated to valuing and acknowledging partners and their contributions

“I am left feeling like my feedback is not appreciated or even used.”—Excerpt from email

In this excerpt, the AYA partner expressed feeling unappreciated for their time contributed to the project and that they were uncertain of the extent to which their efforts had made any impact. During the App design stage, several AYA and family partners spent significant amounts of their own time reviewing storyboards and video scripts and responding to questions about graphic design and content preferences. Not only did this testimonial convey the importance of communicating back to partners about how their input is incorporated, but it suggested that they needed to be better connected and valued as members of the research team.

To value and acknowledge the contributions of AYA and parent partners, researchers became much more committed to “closing the feedback loop”. For example, by reporting back to PFAC at every stage about whether and how their ideas were incorporated, or providing an explanation when their ideas were tabled for a future version of the App (e.g., due to feasibility and timelines of the project). Researchers also developed systems for ongoing and consistent reporting (e.g., detailed meeting minutes, newsletters, bulletins).

Researchers also listened to what partners felt was missing—the more personal or human side to the relationship. Partners shared that working together on research does not mean that AYA and researchers only wish to talk about the research. We identified the importance of having time for an informal conversation during our meetings and we shifted to offering a bulletin of project updates ahead of each meeting to reserve time for discussion. The PFAC also generated their own ideas for opening more direct lines of communication among themselves and with other project team members, such as the clinical partners and staff who joined during stage two of the project with the RCT. We took turns doing “personal shares” at PFAC meetings where all team members had the opportunity to share about themselves including their personal interests, motivations for partnering, and what they hoped to get out of this project. We also invited clinical partners to attend “RCT guest spots” to share about their work about healthcare transition, sparking conversation about potential next steps to enhance healthcare transition with and for youth with disabilities and their families. We invited all team members to complete a “Getting Connected Bio” about themselves that was shared as an indexed document via a private link for quick reference to get acquainted with who was speaking during meetings. Getting to know one another better (e.g., our motivations, interests, personal stories) brought a new energy to the team and helped to facilitate collaboration and networking opportunities, as well as broader conversations during PFAC meetings (e.g., beyond the narrow objective of the project). One outcome of these types of broader conversations was a growing interest among PFAC and other team members, including clinical partners, to better understand decision-making for the “hard transfer” that often occurs at age 18 from the pediatric to the adult healthcare system in Canada. Discussions at PFAC meetings led to our team applying for, and receiving funding from, the 2021 CHILD-BRIGHT Summer Studentship program to support a “policy subgroup” to further explore and advocate on this topic. We scanned grey literature (e.g., websites of hospitals, bills from the Canadian federal and provincial government) on existing policies in Canada to support healthcare transition. We then held a dialogue event to discuss key priorities and suggest policy recommendations for improving the process of healthcare transition, and findings from our work have now been published [30].

### Intention #3: From an imbalanced relationship to one that provided choice and leveraged individual interests and strengths

“It would be awesome for this to grow into more of a two-sided relationship, rather than one-sided.”—Excerpt from email

AYA and parent partners expressed feelings that there was a power imbalance between themselves and other members of the research team. As shared in the AYA partner’s email to the project staff, the AYA partner hoped for the relationship to grow such that researchers, AYA, and parent partners could benefit and be meaningfully involved in project activities. Similar sentiments were expressed during individual check-in meetings with other PFAC members who requested more opportunities to engage in tasks that were of interest to them. Based on this feedback, strategies were implemented to facilitate opportunities for co-leadership where PFAC partners co-led different initiatives. These co-leadership opportunities allowed for power to be shared between PFAC partners and researchers based on the interest of PFAC partners to be actively involved in specific initiatives. We shifted to fewer PFAC meetings (from monthly to quarterly), and we initiated a new approach of working together in subgroups to provide more time and opportunity for AYA and parent partners to engage in specific activities. This shift in meeting frequency allowed for smaller groups to work on different pieces of the project based on tasks they were most interested in. Subgroup tasks were communicated to the PFAC via email. As a matter of routine to help partners decide if they wished to be involved, project staff provided a description of the work and its relevance to the project, as well as an estimate of the anticipated commitment expectations (e.g., number and expected dates of meetings, duration of meetings, expected turn-around for feedback). The creation of subgroups leveraged individual strengths, allowing partners to choose a role for themselves that fit best with their skills and interests. Working in smaller groups also allowed for deeper levels of engagement, and for more hands-on experiences that AYA and parent partners found meaningful.

At the outset of each subgroup task, we used the Involvement Matrix [29] to demonstrate choice and to be intentional in providing opportunities where PFAC members could be engaged as equal partners alongside researchers. Table 1 provides examples of subgroup activities that took place throughout the READYorNot™ BBD Project. Among these include opportunities where PFAC members provided leadership on initiatives that were personally meaningful to them (e.g., development of an advocacy and policy planning perspective paper addressing age of transfer from pediatric to adult healthcare services [30]), co-developed knowledge translation products (e.g., a research video series to explain research in youth-friendly terms) and co-presented at conferences and webinars as well as co-authored research papers.

**Table 1:** Examples of READYorNot™ BBD Project subgroup activities

Subgroup activity	Description	PFAC role
Technical support	To co-create a series of videos to introduce the MyREADY Transition™ BBD App to RCT participants and to describe how to use features of the App for the Technical support website	Reviewed the video scripts, co-created the storyboards and visuals, and provided narration
Research video series	To co-create a series of short videos as an integrated knowledge translation tool to explain research in youth-friendly language, with the READYorNot™ Brain-Based Disabilities Project as an example of an RCT	Provided feedback for the video scripts, co-created the storyboards and visual, and provided narration
E-learning modules	E-learning modules were developed to train and test fidelity of study procedures among project staff at each project site	Co-developed one of the e-learning modules to explain about the importance of patient and family engagement in research, providing feedback on the content and an audio-recording to incorporate into the module
Co-presentations at conferences	PFAC members were invited to co-present at several conferences [32–34]	Brainstormed ideas, provided feedback on abstract submissions, scripted content, provided suggestions for the visuals of the presentation, co-presented live or via pre-recording, answered questions from the audience
Website of resources for youth who were not eligible to participate in the study	A website was developed to: Thank youth and families for their interest in the READYorNot™ Brain-Based Disabilities Study; Share information about the possible reasons that youth and families were not eligible to participate in the study; Provide resources for youth who were not eligible to participate in the study, including resources to other childhood disability research studies, information about the transition from pediatric to adult healthcare, as well as apps and health technology	Reviewed and provided feedback on the language of content and layout of the website, as well as shared potential resources for youth and families
Co-development of an advocacy and policy planning perspective paper	(1) A literature review of existing legislation surrounding the age of transfer in the 4 regions of our trial; (2) Development of patient vignettes to facilitate a dialogue event—with the goal to highlight gaps in the current landscape and to develop recommendations for a future state in terms of transition from pediatric to adult healthcare; and (3) Development of an advocacy and policy planning perspective paper to serve as a stepping stone for improving transition experience for youth with BBD in Canada [30]	Provided significant input in the planning and organization of the dialogue, co-hosted and helped to facilitate breakout room sessions. Shared relevant and real-life examples to help inform the development of a set of patient vignettes and additional prompting questions. Ongoing work to co-author a paper

## Discussion

### Key recommendations and lessons learned

In preparing this case study, our team reflected on our intentions in terms of “where we are now and how far we have come”. At the outset of the project, our team had different expectations about the role of a PFAC and how we would conduct POR. In this section, we bring together our own experiences with examples from the literature which we have found helpful in our POR work together. The critical turning point described herein led to an evolution of learning and growth, including four key recommendations and lessons learned along our research partnership journey: (1) charting the course; (2) learning the ropes, (3) getting all hands on deck, and (4) making course corrections, and being prepared to weather storms. A summary of our strategies that aligns with our four key recommendations are presented in Table 2.

**Table 2:** Recommended strategies for partnerships in research

Key recommendations	Suggested strategies	Additional information (examples, resources, and consideration)
1. Charting the course: Agree upfront on the plan and goals for engagement to guide your work together	Initiate honest conversations upfront to understand preferences for how partners would like to be involved, and how researchers can make partners feel welcome to collaborate throughout the research process Be opened to exploring different roles that adolescents, young adults, and parent partners might prefer, including as co-leads on activities with researchers	Example: Ask partners about preferred communication methods
2. Learning the ropes: Develop capacity for patient-oriented research in adolescents and young adults, parent partners, researchers, and project staff	Allocate resources for comprehensive patient-oriented research training for all members of a research team early in the partnership	Resource: Provide a list of reputable training programs in POR
3. All hands on deck: Build a community of engagement where adolescents and young adults and parent partners feel welcome and supported	Facilitate accessible participation for adolescents, young adults, and parent partners across all stages of the research process Foster ongoing discussions about motivations, goals, skills, and interests with adolescents, young adults, and parent partners Organize activities promoting team bonding on a personal level, such as sharing sessions during meetings, personalized onboarding, and regular check-ins	Consideration: Discuss accessibility measures for virtual participation
4. Making course corrections and being prepared to weather the storms: Remain open to critical reflection, re-evaluation, and adjustment as necessary	Cultivate a willingness to listen, communicate, and make adjustments based on critical reflections and feedback Designate project staff to coordinate, organize, and facilitate partnerships effectively, Incorporate ongoing reflection and evaluation into the partnership process for continuous improvement and adaptation	Consideration: Establish regular channels for communication and feedback

1. *Charting the course*: Agree upfront on the plan and goals for engagement to guide your work together

Team members should work to get on the same page regarding the meaning, purpose, and role of patient-oriented research. The role of AYA and parent partners and the general goal of incorporating POR need to be considered prior to commencing the research project. Two issues that should be considered *prior* to commencing a project are the anticipated roles that AYA and parent partners will hold as members of the research team as well as the overarching goal of incorporating POR. As noted throughout this paper, our research team chose to adopt a PFAC model for engaging AYA and parent partners and held separate meetings for researchers and partners. In hindsight, this structure may not have been optimal, as it created siloes, with some AYA and parent partners feeling as though they were not invited to “the larger table” (e.g., “full” team meetings). Once we recognized this gap, we implemented several strategies; for example, we created a liaison role between the PFAC and the larger RCT team, and we incorporated “guest spots” at PFAC meetings so that members of the RCT team could meet and introduce themselves to PFAC members. However, in future studies we recommend having upfront discussions (i) with AYA and parent partners about how they would like to be involved, and (ii) with researchers about how to make partners feel welcome and how to collaborate on various aspects of the research (a point on which we further expand below). These conversations should begin when the research is first being planned and continue throughout the duration of the project. This is an opportunity for everyone to reflect on what they bring to the project and to articulate their values, expectations, and goals for engagement so that they can effectively work together as a team.

We also reflect on how we proposed to do POR during the first stage of the project, and how our engagement evolved over time. Initially, researchers did not plan for a PFAC, but proposed to engage AYA and parent representatives in more of a paid consultative role (e.g., both as consultants and as participants) in focus groups, interviews, usability/acceptability testing, and on the prototype development team. The plan was well-intentioned in attempting to include AYA and parent perspectives, but it illustrates a baseline naiveté about genuine POR and the distinction between activities involving AYA and parents as research participants (data collection) or as consultants (giving a rubber stamp) versus activities involving them as partners and full members of the research team (engagement). We recognize that a limitation of our partnership is the need to address power imbalances and hierarchy. Researchers were open to hearing about the different roles that AYA and parent partners might prefer, with some activities that they wanted to co-lead with researchers. We recommend having honest conversations guided by discussion and reflection tools [29, 31] at the beginning of the partnership to outline the roles that researchers and partners may prefer to have, including as co-leads and as authentic partners throughout the project.

(2) *Learning the ropes*: Develop capacity for POR in AYA, parent partners, researchers, and project staff

The field of POR is rapidly evolving, with new knowledge and standards developed every year. Education and training are needed for partners and researchers together in an environment that supports trust, respect, reciprocity, and co-learning [32]. We recommend that engagement training early for all members of the project team can help develop capacity in team members and facilitate discussions toward a shared understanding of POR, setting the stage for engagement and interaction throughout the entire project. Prior to beginning this project, the researchers on our team had varying degrees of experience engaging AYA and parents in research; several had none. When our team was formed in 2017, POR training programs and modules that were recommended by our funding body were still being developed. New programs and training modules have since been developed [33–36]. Now that these programs are more widely available, we recommend allocating resources for all members of a research team to engage in POR training as early as possible in the process of partnering. In addition to POR training, research teams may also benefit from reviewing principles of community-based action research, participatory action research, and health equity [37–41]. Having this training would have prepared us for challenges related to engagement that need to be considered throughout a project, providing a shared framework and language for how to consider these challenges.

(3) *All hands on deck: * Build a community of engagement where AYA and parent partners feel welcome and supported

Authentic engagement is about creating a community of engagement where reciprocity is valued and where partners have the opportunity to be involved in ways that are meaningful to them. AYA and parent partners should be invited to contribute to all aspects of the research project, and it is the responsibility of the research team to make participation as accessible as possible.

Partners have reported that the invitation to engage does not automatically lead to collaborative work that is authentic and meaningful, and that it is important to create space for engaging in conversation and supporting contributions beyond the insight of a lived experience [45]. For example, having ongoing discussions with AYA and parent partners about their motivations, goals, skills, and interests when engaging together in research. Harrison et al. [42] suggest it may be beneficial to move beyond focusing solely on research project activities to also include activities that allow teams to get to know one other on a personal level [42]. In our project, we implemented several strategies to get to know one another better (e.g., time during meetings to share more personally, personalized onboarding, regular check-ins). The creation of this time and space allowed for the discovery of shared passions and for opportunities for the PFAC to engage in ways beyond what they “signed up for.” We therefore recommend creating an inclusive, respectful, and welcoming space in research to open the lines of communication for authentically engaging with and empowering AYA and parent partners in ways that are meaningful to them. Several members of our project team co-authored a paper that identified key building blocks in establishing a culture of engagement, including openness to learning from others, a commitment to relationship building, and a drive to grow and improve [43].

Opportunities for engagement should run the full gamut of the research cycle, including later stage activities such as data analysis, interpretation, and dissemination of results based on guidance from institutions and funding bodies such as CIHR [8]. In February 2023, we held a series of four themed collaborative data interpretation meetings where all members of our team (AYA, parent partners, researchers, trainees, and project staff) were invited to a short presentation and a discussion of results. These meetings elicited reflections about the key findings, plans for disseminating and mobilizing results, and invited everyone to think about how they would like to be involved. We talked about criteria for authorship on scientific or scholarly publications, as well as other ways to contribute, including examples of other formats for sharing research results such as infographics, research briefs and webinars. We advocate for at least one AYA and/or parent partner being invited to co-author any publication arising from POR projects, as a matter of course. While working together on our publications, we followed recently established guidelines [44].

Practical strategies to support and operationalize PFAC member engagement in POR include creating an environment where the PFAC members are making a genuine and unique contribution, building community between PFAC members and researchers, best practice activities for researchers to facilitate engagement, and tools and training [42]. These strategies converge and resonate well with our own experience. Strategies that we found especially helpful for promoting and supporting engagement in AYA PFAC members were knowing PFAC members’ skill sets and interests (and aligning them with subgroup tasks), expectation setting, being specific with tasks, and providing information in clear, plain language. For example, we had PFAC members who had a creative interest in creating a study logo or developing storyboards, along with acting and voiceover experience to co-create videos. We also had PFAC members who advocated for non-ableist language in all research materials, as well as members who had social media connections to support participant recruitment.

For research engagement to be authentic and meaningful, we need to develop a relationship based on dignity and respect, set clear expectations, build rapport, have tangible supports, use clear communication, and devote time and space to work together [45]. In our project, clear communication was key to offering choice, and understanding the roles that AYA and parent partners wanted to have in the research process.

(4) *Making course corrections and being prepared to weather the storms*: Remain open to critical reflection, re-evaluation, and adjust as necessary

We can improve the process and progress of engagement by being open and listening, by continuing to re-evaluate and reflect, and by coming together as a team to adjust the goals and procedures as needed. Challenges in the partnership journey have been described by other research teams where a tension was seen as a catalyst to help grow and improve the relationship over time [46, 47]. Good intentions, mutual respect, clarity about roles, a lot of time and flexibility, passion, and sense of humour have led the team to new places in terms of the close collaboration, relevance, and quality of their research. Factors specific to the researcher (e.g., openness of researchers to feedback, liking the researchers) have been reported to be the most frequently reported facilitators to meaningful and active partnership of patients on a research team [20]. Team partnerships can evolve throughout the life of the research program, contingent on the acceptance of tension and willingness to move past it, two‐way communication, willingness to collaboratively identify solutions to problems, and leadership of key team members [47]. We believe that throughout our project, the key to building and maintaining our relationship with the PFAC was grounded in our ability to listen, communicate back (e.g., close the feedback loop), and to make adjustments when necessary. Our ability to pivot in this way can be credited in part to having dedicated project staff to coordinate, organize and facilitate the partnerships. To genuinely partner with patients and families takes time and effort, which would be extremely difficult to do without members of the research team having time dedicated to such efforts.

The recommendations outlined here are congruent with the “best practice” approaches set out in the literature [48], including: train and educate researchers and patients, clarify roles of partners, evaluate the engagement process on an ongoing basis, set and manage expectations/realistic goals, define scope of engagement for each project, consider the Patient & Family Advisory Council (PFAC) model, allow informal socializing/networking, work in small groups, and allow time to build relationships [48]. The two most commonly reported foundational principles were respect and importance of providing training and education for both patient partners and researchers [48]. We recommend that other teams be open to listening to their partners, and to engage in ongoing reflection and evaluation throughout the process of partnering together.

## Conclusions

In research, we often want to present only our best and most polished work; however, by exposing our learning process, we believe there is an even greater opportunity to learn and evolve. In preparing this paper, we chose to be vulnerable regarding genuine challenges that we encountered while partnering, an approach that was appreciated by the project’s AYA with BBD and family partners. As we journeyed together throughout this project, we evolved away from a consultative model of engagement and towards something more collaborative and mutually beneficial. Navigating these changes required a strong commitment of time, resources, and energy from everyone on the project. We believe that our lessons learned can be applicable to other populations outside of youth with disabilities and their families. Our key recommendations align with the current literature, and we hope that other research teams will find our practical guidance beneficial to building and sustaining ongoing partnerships with youth and families in research. To quote a well-known proverb that resonated with our team throughout this experience, we firmly believe that *“If you want to go fast, go alone. If you want to go far, go together”* (unknown origin).

## Data Availability

All data generated or analysed for this study are included in this published article.

## Abbreviations

App: (Digital) application
AYA: Adolescents and young adults
BBD: Brain-based (developmental) disabilities
CHILD-BRIGHT: Child health initiatives limiting disability-brain research improving growth and health trajectories (network)
CIHR: Canadian institutes of health research
e-health: Electronic health
IT: Information technology
PFAC: Patient and family advisory council
POR: Patient-oriented research
RCT: Randomised controlled trial
SPOR: Strategy for patient-oriented research
